# Single-Nucleotide Polymorphism rs7251246 in *ITPKC* Is Associated with Susceptibility and Coronary Artery Lesions in Kawasaki Disease

**DOI:** 10.1371/journal.pone.0091118

**Published:** 2014-03-12

**Authors:** Ho-Chang Kuo, Yu-Wen Hsu, Mao-Hung Lo, Ying-Hsien Huang, Shu-Chen Chien, Wei-Chiao Chang

**Affiliations:** 1 Department of Pediatrics, Kaohsiung Chang Gung Memorial Hospital and Chang Gung University College of Medicine, Kaohsiung, Taiwan; 2 Department of Clinical Pharmacy, School of Pharmacy, Taipei Medical University, Taipei, Taiwan; 3 Department of Pharmacy, Taipei Medical University Hospital, Taipei, Taiwan; 4 Department of Medical Laboratory Science and Biotechnology, Kaohsiung Medical University, Kaohsiung, Taiwan; 5 Department of Pharmacy, Taipei Medical University-Wan Fang Hospital, Taipei, Taiwan; Goethe University, Germany

## Abstract

Kawasaki disease (KD) is a multi-systemic vasculitis that preferentially affects children. A single nucleotide polymorphism (SNP) in inositol 1,4,5-trisphosphate 3-kinase C (*ITPKC*) has been identified to be an important polymorphism in the risk of KD. This study was conducted to comprehensively investigate the associations between all tagging SNPs of *ITPKC* in the risk of KD in a Taiwanese population. A total of 950 subjects (381 KD patients and 569 controls) were recruited. Seven tagging SNPs (rs11673492, rs7257602, rs7251246, rs890934, rs10420685, rs2607420, rs2290692) were selected for TaqMan allelic discrimination assay. Clinical data of coronary artery lesions (CAL) and aneurysms were collected for analysis. A significant association was found between rs7251246 in *ITPKC* and CAL formation. Haplotype analysis for *ITPKC* polymorphisms also confirmed this association in the patients with CAL and aneurysm formation. This is the first study to identify that SNP rs7251246 in *ITPKC* is associated with the severity of KD.

## Introduction

Kawasaki disease (KD) is a systemic inflammatory vasculitis which was first reported in 1974 [1]. It occurs worldwide and mainly affects children less than 5 years of age, and especially in Asian countries. Japan, Korea and Taiwan have the highest reported incidence rates of KD worldwide [2]–[4]. The clinical characteristics of KD include a prolonged fever for more than 5 days, bilateral non-purulent conjunctivitis, diffuse mucosal inflammation, polymorphous skin rashes, indurative angioedema of the hands and feet followed by desquamation, and unilateral non-suppurative cervical lymphadenopathy [3], [5]. The most serious complication of KD is the development of coronary artery lesions (CAL) [6], [7]. In developed countries, KD has become the leading cause of acquired heart diseases in children.

The cause of Kawasaki disease is still unclear, and both genetic and environmental factors are considered to be important in the risk of KD. In 2008, Onouchi et al. first indicated that a functional SNP (rs28493229) in the inositol 1,4,5-trisphosphate 3-kinase C (*ITPKC*) gene was associated with KD susceptibility and the development of CAL [8]. This polymorphism of *ITPKC* (rs28493229) is located in intron 1, and results in different transcriptional levels of mature mRNA by interfering with the efficiency of RNA splicing. In addition, a meta-analysis revealed that the functional polymorphism rs28493229 in *ITPKC* (rs28493229) significantly contributed to the risk of KD [9]. Furthermore, another SNP in *ITPKC* (rs2290692 in 3′UTR) was reported to be associated with the susceptibility of KD in a Han Chinese population [10]. Treatment of refractory KD with a calcineurin inhibitor also highlights the role of an ITPKC-mediated immune system in KD [11].

In this study, we comprehensively examined the association between all tagging SNPs in *ITPKC* and the risk of KD. Seven tagging SNPs (rs11673492, rs7257602, rs7251246, rs890934, rs10420685, rs2607420, rs2290692) were tested. Clinical data of CAL was also evaluated.

## Methods

### Patients Studied

A total of 381 patients with KD and 569 controls were enrolled in this study. The prevalence of KD is less than 1/1,000 children in the general Taiwanese population. Therefore, we assumed that there were no cases of KD in the control group. All of the KD patients were initially treated with a single dose of intravenous immunoglobulin (IVIG) (2 g/kg) during a 12-hour period. This study was approved by the Institutional Review Board of Chang Gung Memorial Hospital, and written informed consent was obtained from either the parents or guardians of the children. The patients whose symptoms did not fit the KD criteria and those who had suffered from an acute fever for less than 5 days were excluded. All of the KD patients underwent 2-dimensional pulse Doppler and color flow imaging at least 3 times within 6 to 8 weeks from the onset of the illness [12], [13]. Two-dimensional echocardiography was performed to visualize the diameter of the right and left coronary arteries on the parasternal short-axis view of the aorta [6]. In accordance with the Japanese Ministry of Health guidelines, a CAL was defined by the internal diameter of the coronary artery being greater than 3 mm (4 mm if the subject was over 5 years of age) or the internal diameter of a segment being at least 1.5 times that of an adjacent segment, as observed in echocardiography. The KD patients with coronary artery ectasia or dilatation which disappeared within the initial 4 weeks after the onset of illness were defined as having transient ectasia and were not judged to have CAL. In addition, coronary arteries were classified on the basis of the presence or absence of aneurysms according to criteria from the JCS Joint Working Group. IVIG responsiveness was defined as defervescence 48 hours after the completion of IVIG treatment and no fever (temperature >38°C) recurrence for at least 7 days after the IVIG treatment with marked improvement or normalization of inflammatory signs.

### DNA Extraction

Blood cells were subjected to DNA extraction by first treating them with 0.5% SDS lysis buffer and then protease K (1 mg/ml) for digestion of nuclear proteins for 4 hours at 60°C. Total DNA was harvested using a Gentra extraction kit followed by 70% alcohol precipitation.

### Genotyping

**Figure 1:**
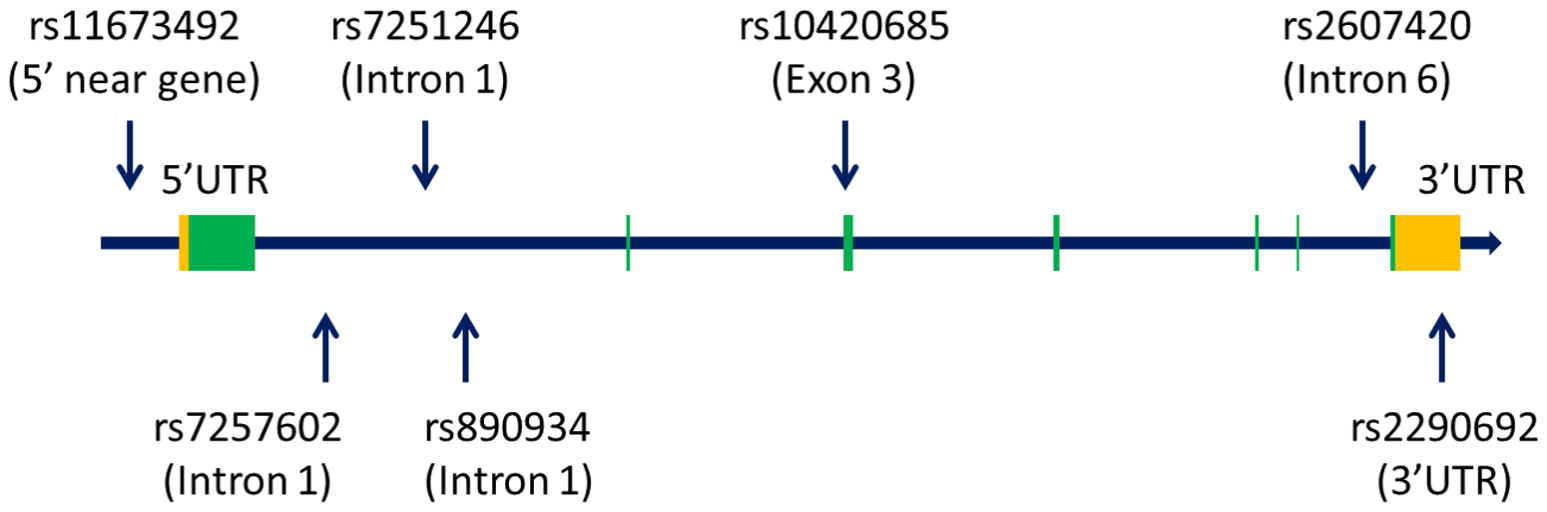
Graphical overview of the *ITPKC* gene polymorphisms.

Seven tagging SNPs (rs11673492, rs7257602, rs7251246, rs890934, rs10420685, rs2607420, rs2290692) with a minimum allele frequency of greater than 10% in the Han Chinese in Beijing population were selected from the HapMap database (http://hapmap.ncbi.nlm.nih.gov/). The *ITPKC* gene structure is shown in **Figure 1**. Genomic DNA was extracted from whole blood samples using the standard method as described previously [14]. Genotyping was carried out using a TaqMan Allelic Discrimination Assay (Applied Biosystems, Foster city, CA). Briefly, polymerase chain reactions (PCR) were performed using a 96-well microplate with an ABI 9700 Thermal Cycler. The thermal cycle conditions were as follows: denaturing at 95°C for 10 minutes, followed by 40 cycles of denaturing at 92°C for 15 seconds and annealing and extension at 60°C for 1 minute. After PCR, fluorescence was measured and analyzed using System SDS software version 1.2.3. The average genotyping success rate in our laboratory is around 95.7%, so some participants did not have genotype data.

### Statistical Analysis

All data are presented as mean ± standard deviation. JMP 9.0 for Windows was used for analysis. Hardy-Weinberg equilibrium was assessed by the χ^2^ test with 1 degree of freedom. The statistical differences between the cases and controls in genotype and allele frequency were assessed by the χ^2^ test or Fisher's exact test. The statistical differences in the genotype and allele frequency in the KD patients with and without CAL or aneurysm formation were assessed using the χ^2^-test. Linkage disequilibrium was assessed for any pair of SNPs, and haplotype blocks were defined using the default setting of Haploview software 4.2 and PHASE version 2.1.

## Results

### Association between *ITPKC* Polymorphisms and the Susceptibility to KD

A total of 381 KD patients and 569 controls were recruited in this study, of whom 66.8% of the cases and 56.0% of the controls were male. The mean ages of the patients and controls were 1.7±1.6 years (± standard deviation (SD)) and 5.7±4.9 years, respectively. Most of the study population was children. Overall, 16.8% (64/381) of the patients with KD had CAL formation (Table 1). The distribution of the *ITPKC* genotypes between the KD patients and healthy subjects is shown in **Table 2**. *ITPKC* SNPs rs11673492 and rs2607420 showed significant associations with KD (recessive model, p = 0.0152 for rs11673492, p = 0.0206 for rs2607420). However, the significance disappeared after multiple testing.

**Table 1:** Characteristics of the patients with Kawasaki disease and normal controls.

	Patients with KD	Normal Controls
Characteristics	N = 381	N = 569
Male gender, No. (%)	247 (66.8%)	314 (56.0%)
Mean (SD) age (years)	1.7±1.6	5.7±4.9
Age range (years)	0–11	0–51
CAL formation	64 (16.8%)	
IVIG resistance	49 (12.9%)	

CAL: coronary artery lesions; IVIG: intravenous immunoglobulin; SD: standard deviation.

**Table 2:** Genotyping and allele frequency of the *ITPKC* SNPs in the controls and patients with Kawasaki disease.

	Genotype	Case (%) (n = 381)	Control Subjects (%) (n = 569)	Allele	Case (%) (n = 381)	Control Subjects (%) (n = 569)	Dominant *P* value	Recessive *P* value	Allelic *P* value
rs11673492	TT	23 (6.2)	60 (10.9)	T	205 (27.7)	342 (31.0)	0.5536	**0.0152** *	0.1250
	CT	159 (43.0)	222 (40.3)	C	535 (72.3)	538 (69.0)			
	CC	188 (50.8)	269 (48.8)						
rs7257602	GG	93 (25.5)	108 (24.7)	G	366 (50.1)	429 (49.0)	0.6280	0.7889	0.6421
	AG	180 (49.3)	213 (48.6)	A	364 (49.9)	447 (51.0)			
	AA	92 (25.2)	117 (26.7)						
rs7251246	CC	74 (19.8)	125 (22.4)	C	345 (46.1)	527 (47.2)	0.8892	0.3396	0.6411
	CT	197 (52.7)	277 (49.6)	T	403 (53.9)	589 (52.8)			
	TT	103 (27.5)	156 (28.0)						
rs890934	TT	72 (22.3)	115 (20.5)	T	290 (44.9)	517 (46.2)	0.1790	0.5386	0.6061
	GT	146 (45.2)	287 (51.3)	G	356 (55.1)	603 (53.8)			
	GG	105 (32.5)	158 (28.2)						
rs10420685	GG	16 (4.3)	25 (4.7)	G	155 (20.8)	222 (21.0)	0.9694	0.7632	0.9387
	AG	123 (33.1)	172 (32.5)	A	589 (79.2)	836 (79.0)			
	AA	233 (62.6)	332 (62.8)						
rs2607420	CC	15 (4.2)	45 (8.1)	C	178 (24.9)	297 (26.7)	0.9211	**0.0206** *	0.3979
	CT	148 (41.5)	207 (37.2)	T	536 (75.1)	815 (73.3)			
	TT	194 (54.3)	304 (54.7)						
rs2290692	GG	73 (19.6)	122 (22.1)	G	340 (45.6)	519 (47.1)	0.8764	0.3475	0.5204
	CG	194 (52.0)	275 (49.9)	C	406 (54.4)	583 (52.9)			
	CC	106 (28.4)	154 (28.0)						

*Significant (*P*<0.05) values are in bold.

### rs7251246 Was Associated With CAL Formation

In the comparison of the distribution of the alleles and the risk of CAL formation, four *ITPKC* SNPs (rs7251246, rs890934, rs10420685 and rs2290692) were observed to be associated with CAL formation. After multiple testing corrections, the significance of rs7251246 remained (**Table 3**).

**Table 3:** Genotyping and allele frequency of the *ITPKC* SNPs in the patients with Kawasaki disease with or without coronary artery lesion formation.

	Genotype	CAL (%) (n = 64)	Without (%) (n = 310)	Allele	CAL (%) (n = 64)	Without (%) (n = 310)	Dominant *P* value	Recessive *P* value	Allelic *P* value
rs11673492	TT	3 (4.8)	20 (6.6)	T	37 (29.8)	167 (27.6)	0.3767	0.5990	0.6209
	CT	31 (50.0)	127 (42.1)	C	87 (70.2)	437 (72.4)			
	CC	28 (45.2)	155 (51.3)						
rs7257602	GG	12 (19.7)	78 (26.2)	G	53 (43.4)	304 (51.0)	0.1597	0.2857	0.1279
	AG	29 (47.5)	148 (49.6)	A	69 (56.6)	292 (49.0)			
	AA	20 (32.8)	72 (24.2)						
rs7251246	CC	14 (21.9)	60 (19.8)	C	71 (55.5)	271 (44.7)	0.0015**	0.7072	**0.0267** *
	CT	43 (67.2)	151 (49.8)	T	57 (44.5)	335 (55.3)			
	TT	7 (10.9)	92 (30.4)						
rs890934	TT	6 (11.8)	62 (23.4)	T	35 (34.3)	245 (46.2)	0.0897	0.0642	**0.0266** *
	GT	23 (45.1)	121 (45.7)	G	67 (65.7)	285 (53.8)			
	GG	22 (43.1)	82 (30.9)						
rs10420685	GG	0 (0.0)	16 (5.3)	G	28 (23.0)	126 (20.7)	0.1532	-	0.5821
	AG	28 (45.9)	94 (30.9)	A	94 (77.0)	482 (79.3)			
	AA	33 (54.1)	194 (63.8)						
rs2607420	CC	1 (1.7)	14 (4.8)	C	34 (28.3)	142 (24.4)	0.1190	0.2729	0.3652
	CT	32 (53.3)	114 (39.2)	T	86 (71.7)	440 (75.6)			
	TT	27 (45.0)	163 (56.0)						
rs2290692	GG	12 (19.0)	61 (20.1)	G	66 (52.4)	271 (44.7)	0.0082**	0.8846	0.1164
	CG	42 (66.7)	149 (49.2)	C	60 (47.6)	335 (55.3)			
	CC	9 (14.3)	93 (30.7)						

*Significant (*P*<0.05) values are in bold. **Significant (*P*<0.01) values are in bold. CAL: coronary artery lesions.

### Haplotype Analysis of *ITPKC* in KD

**Figure 2:**
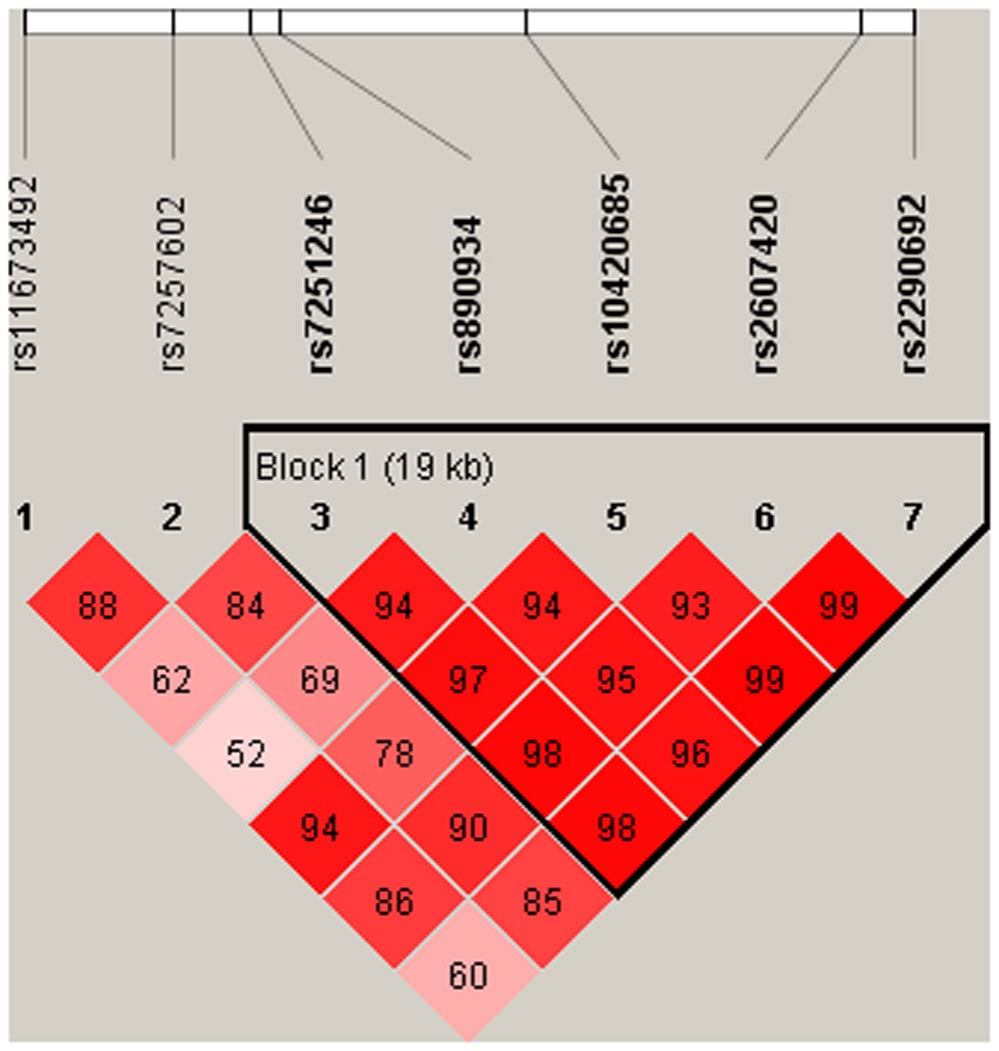
*ITPKC* gene linkage disequilibrium and haplotype block structure in KD. The number on the cell is the LOD score of D'.

We further calculated pairwise linkage disequilibrium (**Figure 2**) and analyzed the haplotypes of *ITPKC* with susceptibility and CAL formation. Pairwise allele analysis indicated that the C/G/G/T/G haplotype had a significant association with CAL formation in the patients with KD compared to the T/T/A/T/C haplotype (*p* = 0.0333, **Table 4**).

**Table 4:** Haplotype frequencies of the *ITPKC* gene in patients with Kawasaki disease with or without coronary artery lesion formation.

rs7251246/rs890934/rs10420685/rs2607420/rs2290692	CAL (%) (n = 64)	Without (%) (n = 310)	OR (95% CI)	*P* value
C/G/G/T/G	25 (26.0)	100 (19.8)	1.87 (1.04–3.34)	0.0333*
C/G/A/C/G	25 (26.0)	116 (23.0)	1.61 (0.90–2.86)	0.1035
T/G/A/T/C	12 (12.5)	54 (10.7)	1.66 (0.80–3.45)	0.1721
T/T/A/T/C	30 (31.3)	224 (44.4)	Reference	

Haplotype frequency less than 1% was excluded. *Significant (*P*<0.05) values are in bold. CAL: coronary artery lesions.

## Discussion

A major advancement in the genetic study of KD was made by the discovery of *ITPKC* in Japan. In 2008, Onouchi et al. first identified a functional polymorphism of *ITPKC* (rs28493229) that was significantly associated with the susceptibility to KD and CAL in both Japanese and US children [8]. Using cell-based functional studies, they further provided evidence to indicate that the risk C allele of *ITPKC* reduced the splicing efficiency of *ITPKC* mRNA that, in turn, possibly contributed to the hyperactivation of Ca^2+^-dependent NFAT pathways in T cells. *ITPKC* is a negative regulator of T cells, and it may function as a calcium channel modulator that is involved in controlling immune systems. Lin *et al.* also reported that the C allele of rs28493229 is associated with KD susceptibility and BCG scar reactivation [15]. Data from meta-analysis studies further support the correlation between rs28493229 of *ITPKC* and susceptibility to KD [9], [14]. In addition, Peng *et al*., tested five *ITPKC* SNPs (including rs28493229, rs79940110, rs10411159, rs2290692, and rs1045705) and found a significant association between rs2290692 and susceptibility to KD [10]. We also confirmed the association between rs2290692 and CAL formation in Taiwanese KD patients. As our findings are in good agreement with the detailed study by Peng *et al.*, we speculate that other genetic variants of the *ITPKC* gene may be associated with KD. Indeed, a novel SNP, rs7251246, located in intron 1 was identified as an important marker of KD in this study. However, the mechanism by which rs7251246 affects the ITPKC gene expression is still unclear. Splicing caused by rs7251246 might be responsible for ITPKC expression. Further studies on the relationship between *ITPKC* polymorphism (rs7251246) and the downstream functional relevance during immune responses should be helpful to understand the etiology of KD.

Several lines of evidence suggest that the pathogenesis of KD is mediated by T cell signaling [16]. Examinations of autopsy tissue have demonstrated infiltration of T cells into coronary arterial walls [17]. A mouse model study that mimicked the CAL of KD suggested that T cells are involved in the development of CAL [18]. The beneficial effects of mizoribine, an inhibitor of lymphocyte proliferation, in a KD animal model have also been reported [19]. *ITPKC* acts as a negative regulator of T cell activation, and the risk allele may result in the activation of the calcineurin-mediated NFAT signaling pathway leading to T cell over-activation. The association of *ITPKC* and KD has been suggested to be the result of defective phosphorylation of inositol 1,4,5-triphosphate (IP_3_), which releases calcium from intracellular stores resulting in a reduced genetic expression of *ITPKC* in carriers of the SNP [20]. Reduced *ITPKC* activity may increase IP_3_ levels, and further trigger *ORAI1*-mediated calcium influx. We also previously identified polymorphisms in the *ORAI1* gene that were associated with inflammatory diseases [21], [22]. Taken together, the results from current studies on *ITPKC* also highlight the therapeutic potential of T cell-mediated signaling in the treatment of KD and CAL formation.

In conclusion, we identified a significant association between rs7251246 and the severity of KD. Although further replication studies in a second population are needed, the current findings, at least in part, reflect that variants in the *ITPKC* gene play an important role in KD.
